# Visualization of subthalamic nucleus on susceptibility weighted imaging and the verification of accuracy by microelectrode recording

**DOI:** 10.3389/fnhum.2025.1559549

**Published:** 2025-05-27

**Authors:** Kaijia Yu, Qi Yao, Yuanyang Wu, Jianyu Li, Lihua Shen, Xiaosu Gu, Zhongzheng Jia, Jiajia Zhang, Jianhong Shen

**Affiliations:** ^1^Department of Neurosurgery, Affiliated Hospital of Nantong University, Nantong, China; ^2^Beijing Institute of Functional Neurosurgery, Xuanwu Hospital of Capital Medical University, Beijing, China; ^3^Department of Neurology, Affiliated Hospital of Nantong University, Nantong, China; ^4^Department of Radiology, Affiliated Hospital of Nantong University, Nantong, China

**Keywords:** susceptibility weighted imaging, subthalamic nucleus, deep brain stimulation, direct targeting, accuracy, microelectrode recording

## Abstract

**Objective:**

To investigate the advantages of susceptibility weighted imaging (SWI) for visualizing the subthalamic nucleus (STN) and to verify the accuracy of this method by microelectrode recordings (MERs) and deep brain stimulation (DBS).

**Methods:**

We included 42 patients with Parkinson’s disease who underwent STN-DBS in our center. The bilateral STN (*n* = 84) was visualized on preoperative 3-T T2-weighted imaging (T2w) and SWI and compared using a 4-point scale. The contrast-to-noise ratio of STN was calculated and compared between two images. The dorsoventral borders of the STN on SWI and T2w were measured and compared using data recorded by intraoperative MERs.

**Results:**

The visualization scores for the STN and contrast-to-noise ratio of STN relative to the zona incerta and substantia nigra were significantly higher on SWI than on T2w images (*p* < 0.05). There was no significant difference in the location of the dorsal and ventral borders of the STN visualized by SWI when compared with MER data (*p* > 0.05). Errors for the dorsal and ventral borders of SWI-STN, as verified by MER (0.56 ± 0.32 mm and 0.72 ± 0.33 mm, respectively) were significantly lower than errors on T2w (0.75 ± 0.33 mm and 0.82 ± 0.45 mm for the dorsal and ventral borders, respectively) (*p* < 0.05).

**Conclusion:**

3-T SWI was a superior method for delineating the STN than conventional T2w. When applying this method, the dorsoventral SWI-STN border coincided reliably with the physiological border determined by MERs. Direct targeting of the STN using SWI can help optimize preoperative target localization, trajectory planning, and postoperative programming.

## 1 Introduction

Parkinson’s disease (PD) is a progressive neurodegenerative disorder characterized by a spectrum of motor symptoms, including resting tremor, rigidity, bradykinesia, postural instability and gait disorders. Deep brain stimulation (DBS) of the subthalamic nucleus (STN) has been shown to effectively ameliorate the motor symptoms of patients with advanced PD (Bronstein et al., 2011). To maximize therapeutic benefits and reduce potential side-effects, it is crucial to target the STN precisely and implant electrodes in the nucleus in a highly accurate manner. Previous research reported that among 28,000 patients undergoing DBS surgery, 15–34% of patients required electrode adjustment due to poor improvements or side-effects (Rolston et al., 2016). The STN can be localized by direct or indirect methods. Historically, indirect targeting of the STN was commonly utilized based on coordinates obtained from brain atlases or by using the red nucleus (RN) as an internal fiducial marker. However, this indirect manner can be subject to bias due to variations in the anatomy and symmetry of the STN between different patients. The development of magnetic resonance imaging (MRI) techniques allowed the STN to be targeted directly because this method improved visualization of the nucleus. Additionally, intraoperative microelectrode recording (MER) and test stimulation are performed in most centers to help identify the optimal target (Polanski et al., 2015).

Advancements in STN neuroimaging rely on two primary approaches: enhancing the field strength and optimizing the novel sequences. Current imaging modalities can be broadly categorized into two groups: spin echo techniques utilizing water molecule precession characteristics and susceptibility-based techniques. While spin echo sequences including T2-weighted (T2w) and T2 fluid attenuated inversion recovery (FLAIR) images are commonly used by neurosurgeons worldwide for targeting the STN, the borders of the STN remain difficult to distinguish from adjacent structures such as the ventromedial substantia nigra (SN) and the dorsolateral zona incerta (ZI) on 3-T T2w and T2 FLAIR (Chandran et al., 2016). In order to better visualize the STN, susceptibility sequences, such as susceptibility weighted imaging (SWI) and quantitative susceptibility mapping (QSM) are considered appropriate to directly target the STN due to their improved capability to visualize iron-rich structures. They can exploit the magnetic susceptibility differences between tissues to acquire better tissue contrast. Previous studies have reported that iron deposition in the STN increases with aging and with the progression of neurodegenerative diseases. Electron clouds and unpaired electrons within metallic elements can determine the magnetic susceptibility properties of different tissues. Different degrees of polarization, such as a paramagnetic susceptibility, and diamagnetic susceptibility arising from different concentrations of iron in deep gray matter nuclei, allow us to delineate the STN from its surrounding structures on SWI and QSM. In addition, susceptibility sequences are sensitive to slow venous blood containing deoxyhemoglobin, and that can enhance visualization of deep cerebral veins and help surgeons to design DBS lead trajectories avoiding veins (Liu et al., 2013; Chandran et al., 2016). It is important that MRI accurately reflects the target if a clinical center relies solely on direct targeting. In our center, we have found QSM more advantageous than T2w for the delineation of the STN and globus pallidus interna. However, it is difficult to fuse T1-weighted (T1w) images, T2w, and computed tomography (CT) in preoperative planning software as the skull is stripped by QSM. Thus, QSM may cause large errors when targeting the STN due to the fusion error associated with different MRI sequences (Yu et al., 2021).

To the best of our knowledge, only four studies have reported the accuracy of SWI with regards to the direct targeting of the STN; these studies reported variable conclusions (Bot et al., 2016; Bus et al., 2018; Budnick et al., 2024; McEvoy et al., 2015). This may be the reason why DBS surgeons have been slow to adopt SWI into their regular practice. The accuracy of SWI when used to target the STN needs to be further verified in order to optimize direct targeting techniques. T2w is the most studied imaging sequence and remains one of the most commonly used for STN targeting, especially in China. Thus, in the present study, we compared delineation of the STN on 3-T SWI and T2w, and used MERs to investigate the accuracy of 3-T SWI for targeting the dorsoventral borders of the STN.

## 2 Methods

### 2.1 Subjects

We included 42 patients (18 males and 24 females; mean age: 67.0 ± 6.1 years) with idiopathic PD who accepted STN-DBS surgery in our center between November 2022 and May 2024. All patients were deemed appropriate candidates for DBS after comprehensive preoperative assessments by professional movement disorder neurologists and neurosurgeons. The study was approved by the Hospital Ethics Committee, and all patients provided written informed consent prior to MRI and surgery.

### 2.2 Imaging parameters

Preoperative MRI examinations, including 3-T axial T1w, three-dimensional T2w, and coronal SWI, were performed on a 3-T MR scanner (Discovery MR750 3 T, GE Healthcare, Wiscomson, USA) several days prior to surgery. The parameters for 3-T T1w images were as follows: repetition time/echo time: 7.6/2.7 ms; bandwidth: 162.8 Hz; acquisition matrix: 256 × 256; slice thickness: 1 mm; flip angle: 12°; field-of-view: 256 mm; total slices: 312. For 3-T T2w images, the parameters were as follows: repetition time/echo time: 2500/75.9 ms; bandwidth: 244.1 Hz; acquisition matrix: 256 × 256; slice thickness: 1 mm; flip angle: 90°; field-of-view: 256 mm; total slices: 162. For 3-T SWI images, the parameters were as follows: repetition time/echo time: 80.0/43.6 ms; bandwidth: 162.8 Hz; acquisition matrix: 384 × 256; slice thickness: 2 mm; flip angle: 15°; field-of-view: 256 mm; total slices: 212.

### 2.3 Surgical procedure

On the day of surgery, CT scans (slice thickness: 1 mm; slice spacing: 0; Siemens, Erlangen, Germany) were obtained from each patient with their head mounted in a conventional stereotactic Leksell frame (Elekta, Stockholm, Sweden). The neurosurgeon then imported preoperative MR and CT images into Leksell SurgiPlan software (Elekta, Stockholm, Sweden) and fused the preoperative MRI images with the CT scans using rigid-body co-registration. The STN was visually selected on SWI using an anterior commissure/posterior commissure-based coordinate system in SurgiPlan software. The image layer with the largest STN volume was selected on the coronal SWI, and the target was set at the ventral border of the STN where it met the SN. Trajectories were designed through an appropriate gyrus, passing through the dorsolateral sensorimotor area of the STN, and avoiding the intracranial vessels and the ventricular walls. Surgery was performed with the patient in a semi-sitting position and under local anesthesia during lead implantation. The left electrode was usually implanted first, followed by the right electrode. Following burr-hole craniotomy, we placed a rigid guiding tube and acquired MERs through the central channel of a multielectrode holder (Bengun, Alpha Omega, Nazareth, Israel) to verify the electrophysiological border of the STN. Following lead placement (L301, PINS, Beijing, China), we performed test stimulations to test the therapeutic effect and map stimulation-induced side-effects. Subsequently, a rechargeable pulse generator (G102R, PINS, Beijing, China) was placed in a subcutaneous pocket under general anesthesia. After surgery, the CT data was scanned to rule out complications such as intracranial hemorrhage, and then fused with preoperative MRI to verify the lead position, and to measure the deviation of the final lead position from the planning tracks from the perspective of trajectories. The stimulator was turned on 3 weeks after surgery, and the parameters were adjusted over the following months. Postoperative assessments were performed 6 months after surgery using the Unified Parkinson’s Disease Rating Scale part III (UPDRS-III).

### 2.4 Data acquisition

#### 2.4.1 Qualitative analysis

**Figure 1:**
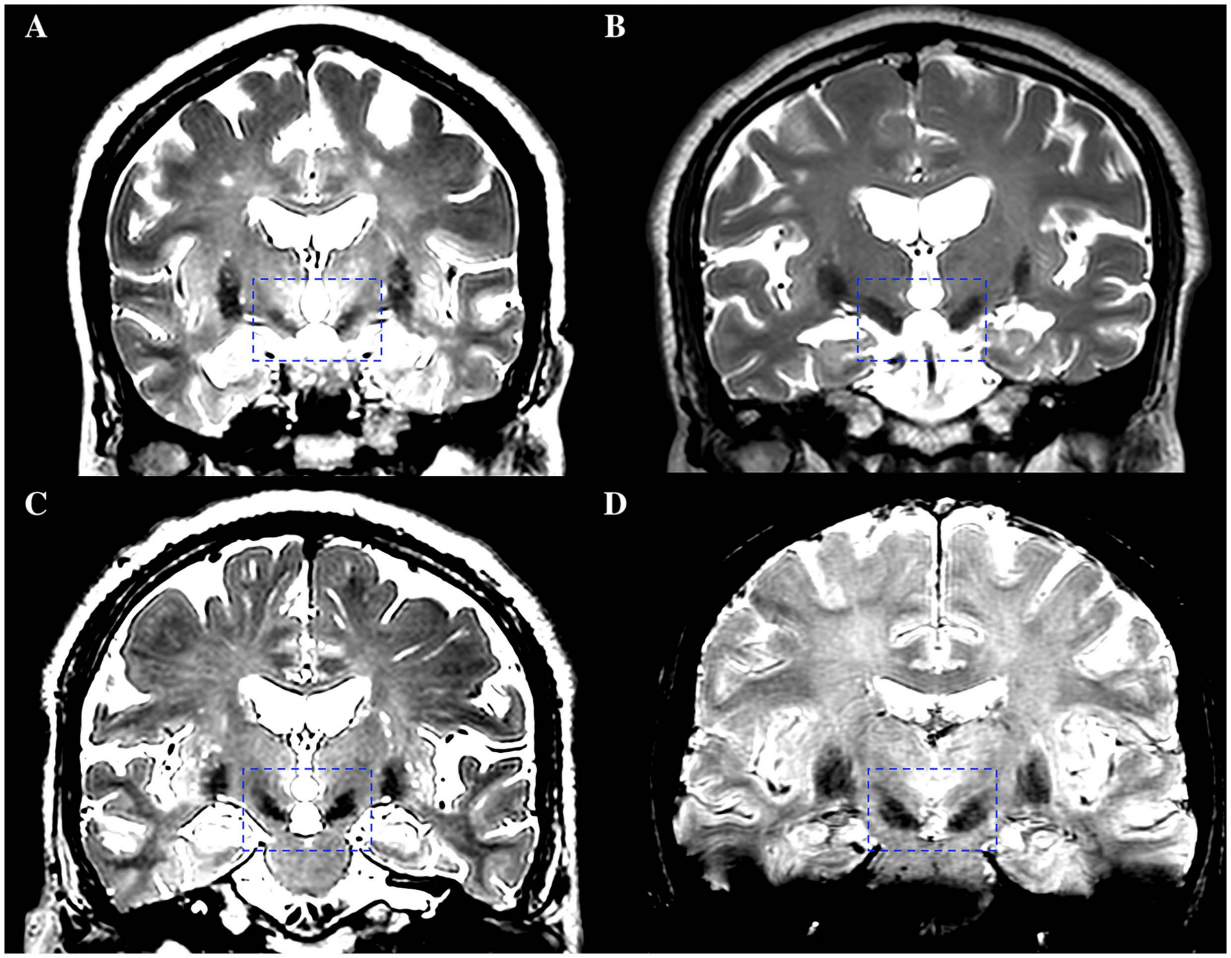
A 4-point scale based on demarcation of STN borders for the visualization scores of the STN. **(A)** Score 0, STN was not visible. **(B)** Score 1, the STN-SN complex was visible with vague borders. **(C)** Score 2, the STN could be clearly differentiated from the dorsal ZI but not from the ventral SN. **(D)** Score 3, the STN was clearly visible with an obvious border with a dorsal neighbor (ZI) and a ventral neighbor (SN). STN, subthalamic nucleus; SN, substantia nigra; ZI, zona incerta.

Two experienced neurosurgeons (YKJ and SJH, with 9 and 29 years of neurosurgery experience, respectively) independently evaluated the visibility of the bilateral STNs on T2w and SWI images. The visualization scores for the STN were graded by a 4-point scale based on demarcation of nuclei borders, as follows: Score 0: STN was not visible (Figure 1A); Score 1, the STN-SN complex was visible with vague borders (Figure 1B); Score 2, the STN could be clearly differentiated from the dorsal ZI but not from the ventral SN (Figure 1C); Score 3, the STN was clearly visible with an obvious border with a dorsal neighbor (ZI) and a ventral neighbor (SN) (Figure 1D) (Liu et al., 2013; Dimov et al., 2020). Discrepancies were resolved by negotiation or by consultation with a third neurosurgeon (YQ). The scores given by the two neurosurgeons were then averaged and recorded.

#### 2.4.2 Quantitative analysis

**Figure 2:**
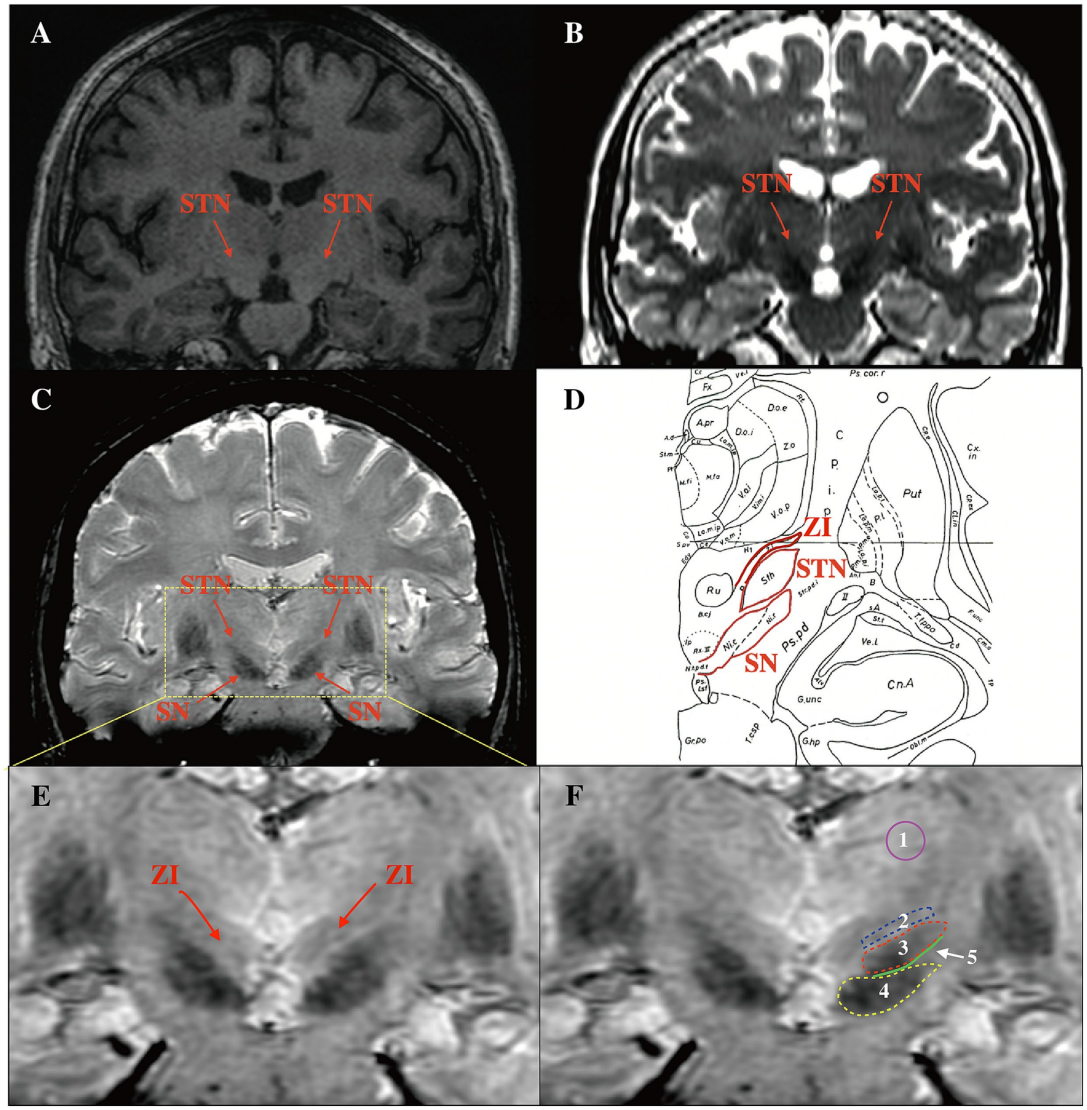
Comparison of the delineation of the STN in different coronal sequences and ROI selection on SWI. **(A)** The STN could not be visualized on T1w. **(B)** The STN and SN were hypo-intense on T2w, but the dorsoventral borders of the STN were difficult to distinguish. **(C)** The STN was hypo-intense on SWI, and the dorsoventral borders were visible. **(D)** Use of the Schalterbrand atlas (plate 27 Fp 3.0) to display relationships between the STN and surrounding structures. **(E)** The ZI was visible on SWI. **(F)** ROI selection. 1: thalamus area 2: ZI 3: STN 4: SN 5: the junction area between the STN and the SN. STN, subthalamic nucleus; ROI, regions of interest; SWI, susceptibility weighted imaging; SN, substantia nigra; ZI, zona incerta; T1w, T1-weighted; T2w, T2-weighted.

To compare the contrast-to-noise ratios (CNRs) of the STN on T2w and SWI, bilateral regions of interest (ROIs), including the STN, SN, and ZI, were drawn manually on the sequences using Horos software (a free and open-source code software program that is distributed free of charge under the LGPL license at Horosproject.org and sponsored by Nimble Co LLC d/b/a Purview in Annapolis, MD USA) (Figure 2F). The CNRs of the STN on T2w and SWI sequences were then calculated using the following equation: CNR = (SI_STN-_SI_ZI/SN_)/*σ*, where SI represents the mean signal intensity of the ROI, the subscript represents the measurement area, and σ represents the background noise (expressed as the standard deviation of the signal intensity in the thalamus area). CNR represents the tissue contrast between the ROI and other surrounding structures. The larger the CNR value, the greater the signal difference between structures, and the easier it is for the ROI to be identified by the naked eye.

#### 2.4.3 MRI and the STN border as defined electrophysiologically

**Figure 3:**
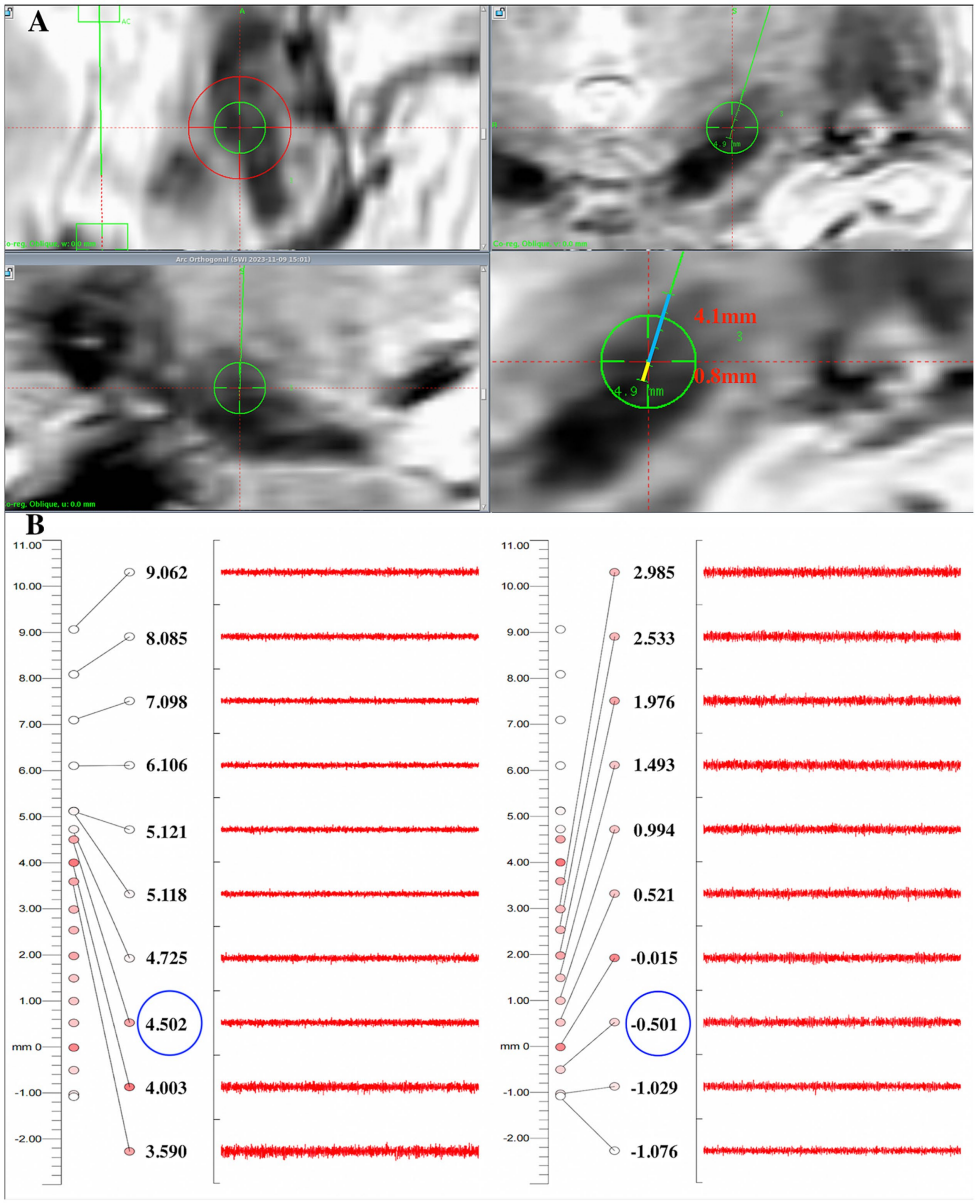
Comparison of the dorsoventral SWI-STN borders with results recorded by electrophysiology in one patient. **(A)** On coronal SWI, the dorsal border of the STN was 4.1 mm away from the target (blue line), and the ventral border of the STN was 0.8 mm away from the target on the planned trajectory (yellow line); **(B)** The MER results showed that the microelectrode entered the dorsal border of the STN about 4.5 mm above the target and exited the ventral border of the STN about 0.5 mm below the target, indicating that the error of the dorsal and ventral borders between SWI-STN and MER-STN was 0.4 mm and 0.3 mm, respectively. STN, subthalamic nucleus; SWI, susceptibility weighted imaging; MER, microelectrode recording.

Window width and levels were adjusted manually to acquire optimal visualization of the STN relative to surrounding structures on each T2w and SWI image. For SWI, a level of 248 and a width of 40–60 was chosen; for T2w, we selected a level of 500 and a width of 190–220. The STN appeared as a hypo-intense structure located anterolateral to the RN on both T2w and SWI images. The substantia nigra is located ventral to the STN, and the zona incerta is located dorsal to the STN on coronal slices. We measured distances of dorsal border of STN to the target (blue line) and ventral border of STN to the target (yellow line) on the trajectory on coronal T2w and SWI images as the MRI-STN border (Chandran et al., 2016) (Figure 3A). Intraoperative MERs commenced 10 mm above the target point, and the microelectrodes were manually advanced with steps of 0.5 mm. When the microelectrode entered the STN, background noise could increase and occasional bursts of an irregular discharge pattern can be observed. The neuronal activity ceased when the microelectrode left the STN, usually with 0.5–1 mm of electrical silence, followed by the recording of high-frequency activity from the SN. Recordings were extended 2–3 mm beyond the target point until STN activity ceased or SN signals were measured (Guridi et al., 2000). We defined the onset of typical STN activity by MER as the electrophysiological beginning of the STN on the current trajectory and continued the recording trajectory until the signals of the STN ceased, which we defined as the electrophysiological end of the STN. The location of the microelectrode entering and leaving the dorsoventral borders of STN can be recorded as the MER-STN border. (Figure 3B).

### 2.5 Statistical analysis

SPSS 26.0 software (SPSS, Inc., Chicago, IL, USA) was used to perform statistical analysis. All data are reported as means ± standard deviations. The Wilcoxon rank-sum test was used to compare visualization scores and the CNR of the STN between SWI and T2w images. Paired sample t-tests were used to compare differences between the dorsal and ventral borders of the STN on SWI and T2w images and the recorded electrophysiological borders. The Chi-squared test was used to compare the number of tracks for a border discrepancy of ≤1 mm and 1–2 mm between the MRI-STN and the MER-STN borders on SWI and T2w images. Statistical significance was defined as a *p* value <0.05 if not specifically stated.

## 3 Results

Typical STN activity was recorded for all 42 patients including 84 tracks during surgery. All patients achieved the amelioration of tremor, rigidity, or bradykinesia during test stimulation. The deviation of the final lead position from the preoperative planning tracks from the perspective of trajectories was 0.63 ± 0.23 mm following the fusion of postoperative CT with preoperative MRI. Intracranial hemorrhage did not occur in any of the patients, although two patients developed obvious pneumocephalus. Patients achieved an improvement of 51.5% ± 12.6% on the UPDRS-III with STN on-stimulation versus off-stimulation in an off-medication state at 6 months postoperatively.

Figure 2D shows a coronal image from the Schalterbrand atlas (plate 27 Fp 3.0) which can be call out on the SurgiPlan software to help recognize the different nuclei and the anatomical relationship of the nuclei to the surrounding structures. We were unable to observe the STN and SN on T1w images (Figure 2A). On T2w and SWI images, both the STN and SN appeared hypo-intense (Figures 2B,C). It is noteworthy that a more superior delineation of the STN relative to the surrounding ZI and SN was provided by SWI images when compared to T2w images. The border of the STN was difficult to distinguish from the ventral SN and dorsal ZI on T2w images. In addition, the ZI was visible on SWI as slight hypo-intensity (Figure 2E); this was not evident on T2w images.

**Figure 4:**
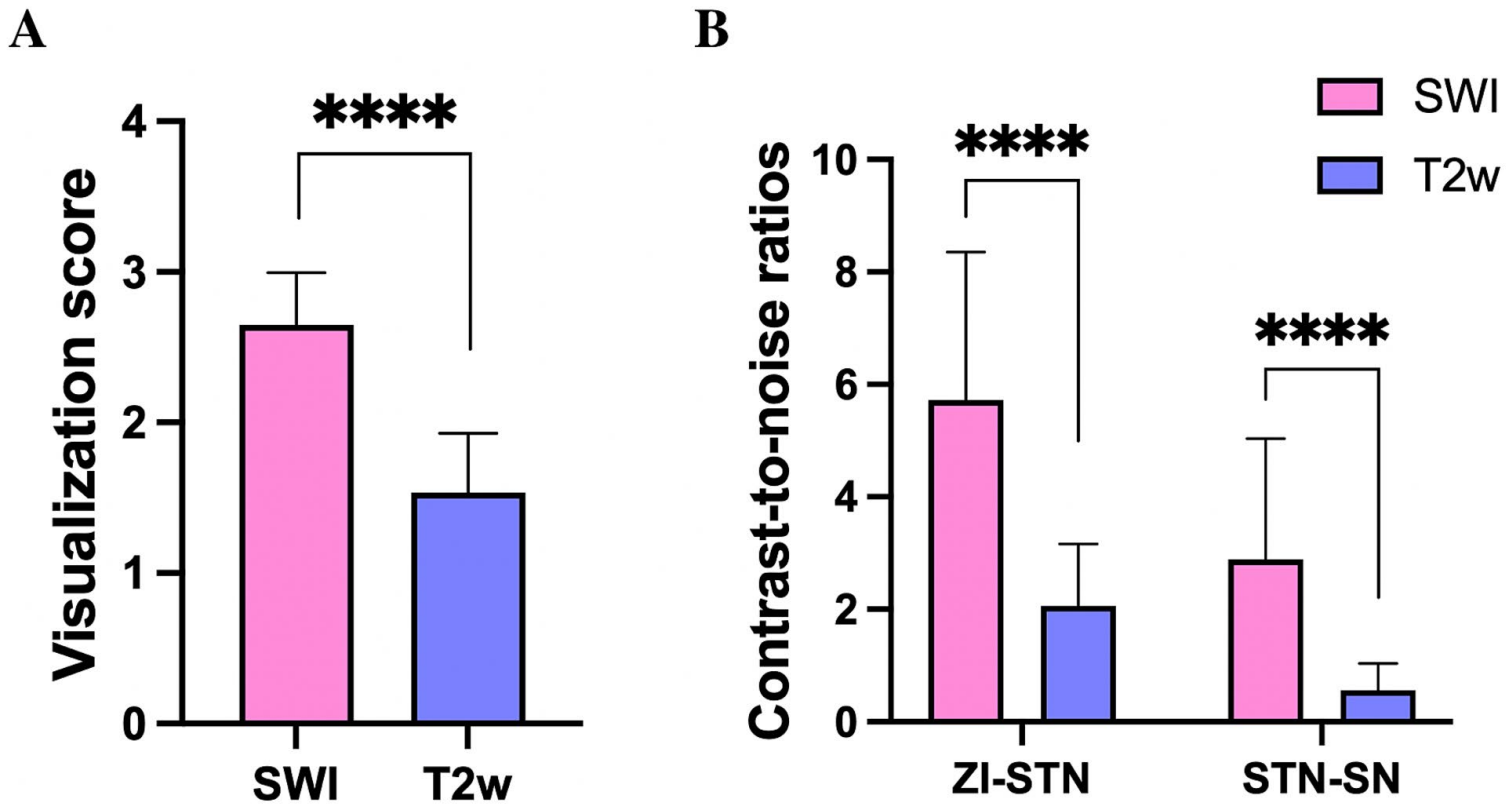
The visualization score for the STN **(A)**, and the contrast-to-noise ratio of the STN relative to the ZI and SN **(B)** on SWI, were significantly larger than those derived from T2w, **** *p* < 0.0001. STN, subthalamic nucleus; SN, substantia nigra; ZI, zona incerta.

Qualitative analysis revealed that the mean visualization scores for bilateral STNs (*n* = 84) on T2w and SWI were 1.5 ± 0.4 and 2.7 ± 0.3, respectively. These scores were significantly different (*p* < 0.05), thus indicating that the STN was more clearly delineated on SWI than on conventional T2w (Figure 4A). The CNRs of the STN relative to the ZI and SN were 5.7 ± 2.6 and 2.9 ± 2.1 on SWI, and 2.1 ± 1.1 and 0.6 ± 0.5 on T2w. The CNR values of STN-ZI (STN relative to ZI), STN-SN (STN relative to SN) calculated on SWI images were 2.7- and 4.8-fold higher than the CNRs calculated on T2w images (*p* < 0.05) (Figure 4B). These data indicated that the STN could be better distinguished from the surrounding ZI and SN on SWI than on T2w due to tissue contrast.

**Figure 5:**
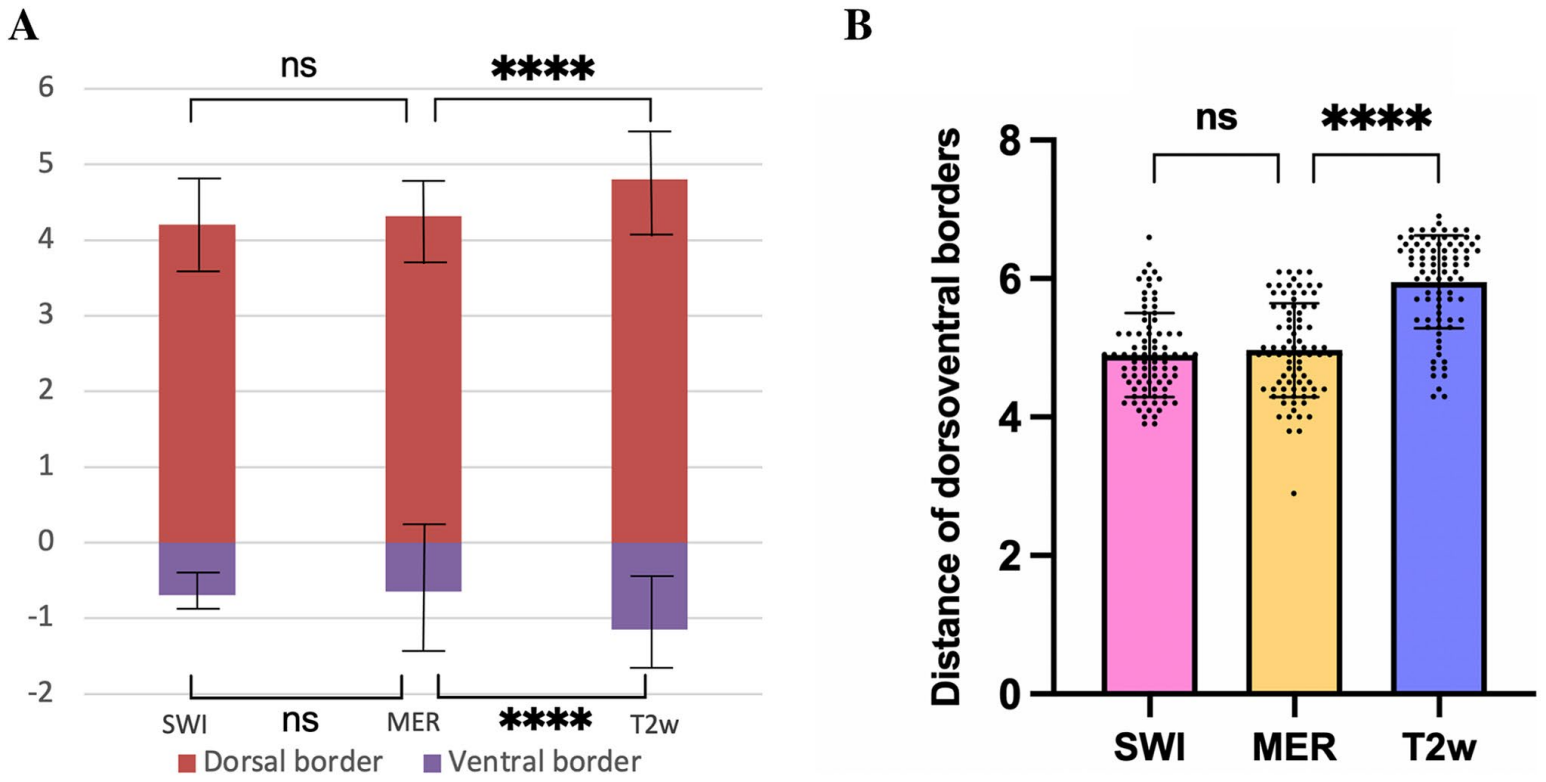
Comparison of the dorsal and ventral borders of the STN **(A)**, and distance of dorsoventral borders **(B)** on SWI, T2w, and recorded by MER. **** *p* < 0.0001, ns indicates no significant difference. STN, subthalamic nucleus; SWI, susceptibility weighted imaging; T2w, T2-weighted; MER, microelectrode recording.

On SWI and T2w, the dorsal border of the STN was 4.2 ± 0.6 mm and 4.8 ± 0.7 mm above the target, respectively; the ventral border was 0.7 ± 0.3 mm and 1.1 ± 0.6 mm below the target, respectively. The dorsal border of the STN recorded by MERs was 4.3 ± 0.5 mm above the target, and the ventral border was 0.6 ± 0.9 mm below the target. There was no significant difference in the location of the dorsal and ventral borders of the STN visualized on SWI when compared MER data (*p* > 0.05). The dorsal and ventral borders of the STN visualized on T2w were significantly larger than the borders recorded by MER (*p* < 0.05) (Figure 5A). These results suggested that the starting and ending position of SWI-STN was consistent with the position of the MER-STN. However, the starting position for T2w-STN was more dorsal than for MER-STN, and the end position for T2w-STN was more ventral than for MER-STN. The total length of the path through the dorsoventral borders of the STN was 6.0 ± 0.7 mm on T2w and 4.9 ± 0.6 mm on SWI. The length of the dorsoventral borders of the STN recorded by MERs was 5.0 ± 0.7 mm. The path length passing through the dorsoventral border of the STN on SWI was comparable to that recorded by MERs (*p* > 0.05), and the path length through the STN on T2w was significantly larger than that recorded by MERs (*p* < 0.05) (Figure 5B).

**Figure 6:**
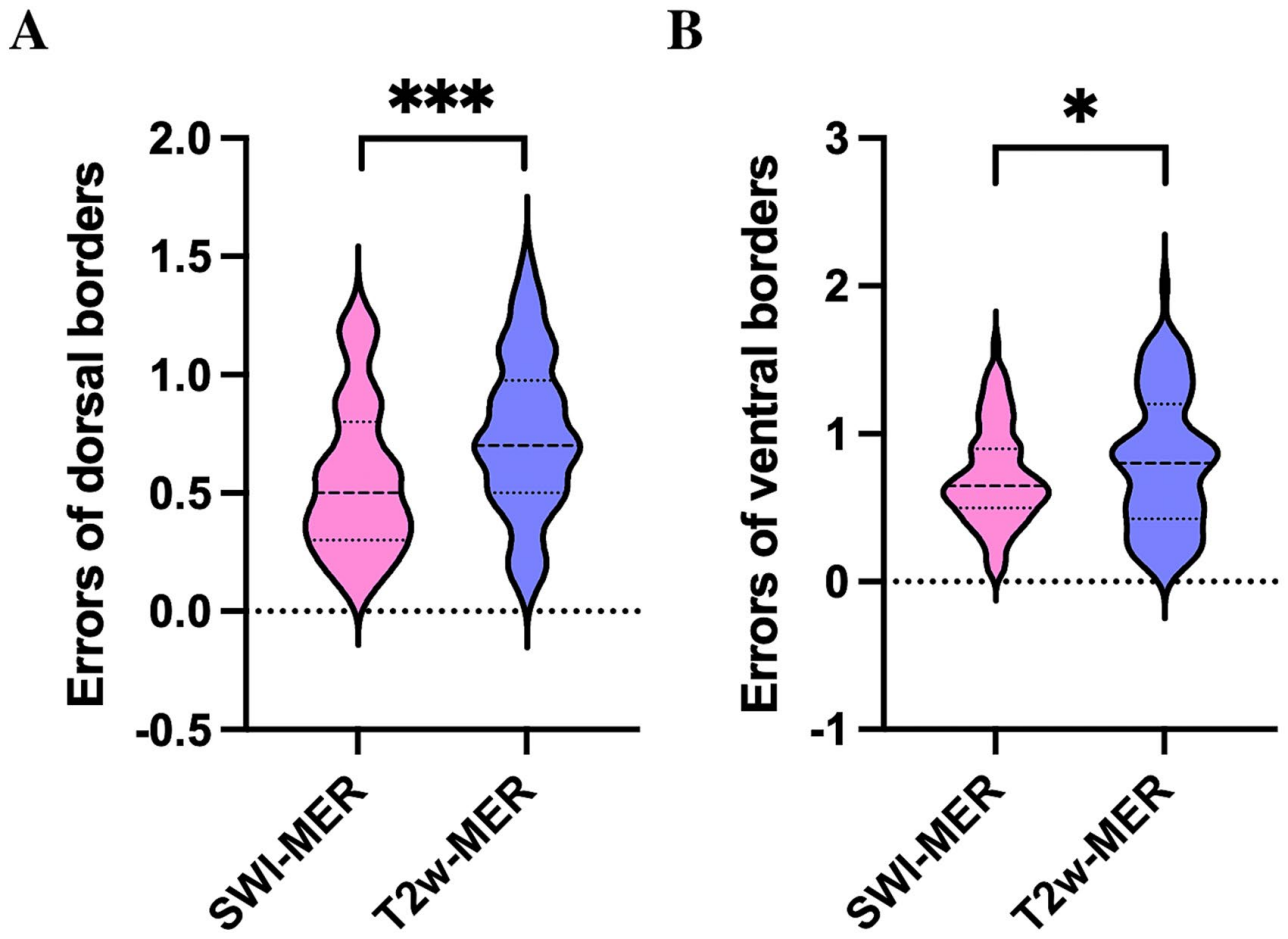
Comparison of errors for the dorsal **(A)** and ventral borders **(B)** of SWI-STN relative to the MER-STN with errors for the dorsal and ventral borders of SWI-STN relative to the MER-STN. *** *p* < 0.001, * *p* < 0.05. STN, subthalamic nucleus; SWI, susceptibility weighted imaging; MER, microelectrode recording.

Differences of the dorsal MER-STN and MRI-STN borders were 0.56 ± 0.32 mm on SWI and 0.75 ± 0.33 mm on T2w. We divided the border error, as measured by MER and MRI, into two groups: ≤ 1 mm and 1–2 mm. When considering individual tracks, the proportion of border errors ≤ 1 mm between the dorsal MRI-STN and MER-STN borders were 86.9% (73/84) on SWI and 77.4% (65/84) on T2w. Chi-squared analysis revealed that there was no statistical difference in the number of tracks with an error of ≤1 mm when compared between the dorsal MRI-STN and MER-STN borders on SWI and T2w (*p* > 0.05). Differences of the ventral MER-STN and MRI-STN borders were 0.72 ± 0.33 mm on SWI and 0.82 ± 0.45 mm on T2w. Errors for the dorsal and ventral borders of SWI-STN relative to the MER-STN were significantly smaller than the errors for the dorsal and ventral borders of T2w-STN relative to MER-STN (*p* < 0.05) (Figures 6A,B). The proportion of border errors ≤1 mm between the ventral MRI-STN and MER-STN borders were 81.0% (68/84) on SWI and 73.8% (62/84) on T2w. Chi-squared analysis indicated that there was no statistical difference in the number of tracks with an error of ≤1 mm when compared between the ventral MRI-STN and MER-STN borders on SWI and T2w (*p* > 0.05). This suggested that the error when directly targeting the dorsoventral borders of STN on SWI, as verified by MER, was not larger than the error when directly targeting borders of the STN on T2w.

## 4 Discussion

STN-DBS has been shown to effectively improve the motor symptoms of patients with PD. Over recent years, STN-DBS has also been used to treat drug-resistant epilepsy with epileptogenic foci located in the sensorimotor area of the cerebral cortex (Yan et al., 2022). The key to successful DBS surgery lies in the accurate placement of electrodes in the sensorimotor area of the STN. Two methods are usually utilized to target the nucleus: indirect and direct targeting. However, due to anatomical differences between individuals, the indirect targeting method, which acquires target coordinates based on a known atlas is not an optimal method for guiding the placement of electrodes. The direct targeting of the STN requires the distinct visualization of the STN borders on MRI; this method depends heavily on image quality.

At present, the sequence commonly used in clinical practice to target the STN is 3-T T2w imaging; however, 3-T T2w cannot clearly visualize all borders of the STN, especially the ventral border which is adjacent to the SN. Thus, conventional coordinates for the STN, as provided by an atlas, and slight adjustments of coordinates according to the relationship of STN with the location of RN are utilized to target the STN in most centers (Andrade-Souza et al., 2005). Ultra-high-field strength MRI techniques, such as 7-T or 9.4-T T2w images, can help to clearly delineate the STN due to improved spatial resolution and tissue contrast (Abosch et al., 2010; Massey et al., 2012). However, image distortion can occur with the enhancement of field strength, thus influencing the precise anatomical targeting of the STN. SWI is a potential imaging sequence for targeting the STN due to its distinct visualization of iron-rich structures and its clear depiction of veins. However, few studies have investigated the accuracy of SWI in locating the STN; furthermore, these existing studies have led to conflicting findings (McEvoy et al., 2015; Bot et al., 2016; Budnick et al., 2024; Bus et al., 2018). The observed discrepancies in clinical study conclusions may be attributed to institutional variations in both DBS surgical protocols and error quantification methodologies. Multiple technical factors potentially impact lead placement precision, including: (1) heterogeneity in imaging sequence quality for STN visualization; (2) algorithmic disparities in image fusion accuracy across DBS planning platforms (Burke et al., 2021) (3) divergent targeting approaches for STN localization; (4) variability in intraoperative imaging modalities for electrode position verification; and (5) inherent mechanical limitations of stereotactic systems. Current evidence suggests that these cumulative technical variables may introduce up to 2 mm of three-dimensional spatial error in stereotactic targeting accuracy (van den Munckhof et al., 2021). In the present study, we investigated the accuracy of SWI by comparing the dorsal and ventral borders of the STN visualized on SWI and T2w images with the electrophysiological borders defined by MER in our center.

In this study, the visualization scores for the STN on SWI were significantly higher than the scores determined on T2w. This suggested that SWI can delineate the dorsoventral borders of the STN better than T2w. In addition, the CNR of the STN-ZI and STN-SN were significantly higher than those measured on T2w, thus indicating that SWI images provide better tissue contrast for the STN relative to surrounding structures than T2w images. This demonstrated that 3-T SWI allowed us to better distinguish the STN from surrounding structures than T2w images, as reported in previous studies (Liu et al., 2013). SWI utilizes magnetic susceptibility differences between tissues to generate images. Thus, the paramagnetic iron in the STN of elderly patients with PD provides good contrast between the STN and its surrounding structures, and makes the STN appear darker on SWI when compared with T2w.

Previous studies have reported that inconsistencies exist between the STN borders when estimated from T2w images, histology, and by MER. Distortion remains one of the most significant challenges in MRI imaging of the STN (Cuny et al., 2002; Dormont et al., 2004; Hamani et al., 2005). This distortion is particularly notable at the peripheries of the target structures because the low tissue contrast between the STN and its surrounding structures in T2w can obscure the borders of the STN (Menuel et al., 2005). It is important to understand how SWI can delineate the border of the STN as this can directly influence the accuracy of nuclei targeting. In our study, we discovered that the locations of the dorsal and ventral borders of the STN when visualized by SWI were consistent with the borders determined by MER (Figure 5A). However, the starting position for T2w-STN was more dorsal than for MER-STN, and the end position of T2w-STN was more ventral than for MER-STN. In addition, the total length of the path passing through the dorsoventral borders of the STN on SWI was consistent with those acquired from MER, although the length of the dorsoventral borders on T2w was significantly larger than from MER. This indicated that the dorsal and ventral SWI-STN borders exhibited high correspondence with the MER-STN. It has been established that non-local magnetic susceptibility effects (blooming artifacts) of SWI may blur the borders of the STN and can theoretically cause them to appear larger than they really are (Chandran et al., 2016). This is why different centers have investigated the accuracy of this technique, and also why this technique has not been used routinely in clinical practice to localize the STN. It is interesting that the size of the STN when visualized on SWI images is smaller than that of the STN on T2w in our study; these findings concurred with those arising from previous studies. One possible reason for this is the limited visual resolution of the naked eye. The fuzzy borders of the STN on SWI is difficult to identify with the naked eye. Furthermore, the adjustment of MRI window width and window level can also significantly influence the size of the STN outline when visualized. When using the RN on T2w as a reference to target the STN, we found that reducing the window level and increasing the window width made the STN and the SN darker on T2w. This allowed the outlines of the STN and the SN to be seen, but their outline will also be larger. The borders of STN and SN were almost clear on SWI images and we did not need to over-adjust the window width and level to achieve better tissue contrast between the STN and surrounding structures to make the STN more hypo-intense. This may be the reason why the dorsoventral borders of the STN when visualized on SWI were smaller than that on T2w.

In addition, the errors of the dorsal and ventral borders of SWI-STN (0.56 ± 0.32 mm and 0.72 ± 0.33 mm, respectively) and T2w-STN (0.75 ± 0.33 mm and 0.82 ± 0.45 mm, respectively) were both less than 1 mm when verified by electrophysiology. The errors of the dorsal and ventral borders of SWI-STN relative to the MER-STN were significantly smaller than the errors of the dorsal and ventral borders of T2w-STN relative to the MER-STN (*p* < 0.05). Moreover, the number of tracks with a border error ≤1 mm when measured by MER and MRI did not differ significantly on SWI and T2w for both the dorsal and ventral borders of the STN. This indicated that direct targeting of the dorsoventral borders of the STN using SWI exhibited reliable accuracy, which was no worse than for T2w.

In this study, the error between the dorsal MRI-STN and MER-STN borders was < 1 mm in 86.9% of the tracks, and the error between the ventral MRI-STN and MER-STN borders was <1 mm in 81.0% of the tracks. This suggested that the dorsoventral SWI-STN and MER-STN borders coincided in a reliable manner, as reported previously by McEvoy et al. (2015). These authors reviewed 28 MER tracks in seven patients and found that in 85.7% of the tracks, the error between the ventral MER-STN border and the ventral border delineated on SWI was <1 mm, concluding that SWI could accurately estimate the electrophysiological border. Similarly, Polanski utilized MER to study the accuracy of STN localization on 3-T SWI, T2w, and FLAIR sequences; these authors reported the higher accuracy of STN targeting based on 3-T SWI when compared with other sequences (Polanski et al., 2015). In addition, Hailey found that SWI closely approximated the electrophysiological borders of the STN, more accurately predicting the dorsal boundary. However, the ventral boundary on SWI was inaccurate compared with the electrophysiological boundary. The quality of SWI may influence the ventral boundary delineated on SWI. They performed SWI imaging with an axial slice thickness of 2 mm due to local limitations, which may affect the dorsal and ventral borders of STN delineated on coronal and sagittal slices (Budnick et al., 2024). Bus et al. reported that the average difference in the ventral border of the STN when measured by MER and SWI was 2.1 mm, and a difference of ≤1 mm was only detected in 33% of tracks (Bus et al., 2018). The low degree of correspondence with the electrophysiological STN may be related to the different methods used. These authors reconstructed the MER tracks using an intraoperative O-arm; fusion of O-arm images and preoperative MRI is inherently known to be associated with certain errors. In addition, MER tracks required further refinement in many of the cases in this previous study; furthermore, these authors analyzed these suboptimal tracks, which could have influenced the ability to accurately target the STN using SWI verified by electrophysiology. Bot et al. studied the accuracy of 1.5-T SWI to visualize the medial and lateral borders of the STN by MER (Bot et al., 2016). These authors found that 21% of the 165 tracks that recorded typical STN electrical activity were located outside the STN outline displayed by SWI. Therefore, these authors considered that SWI could not accurately display the lateral part of the STN. However, the accuracy of 3-T SWI in delineating the medial and lateral borders of STN has yet to be reported.

The clear and reliable delineation of STN borders by 3-T SWI can realize direct targeting techniques. This can help optimize preoperative target localization, trajectory planning, and postoperative DBS programming. In practice, identifying the dorsal and ventral borders of the STN on coronal images is particularly important in DBS. The STN is divided into three functional areas: the dorsolateral sensorimotor area, the intermediate associative area and the ventromedial limbic area. The placement of electrodes in the dorsolateral sensorimotor area of the STN can effectively improve the motor symptoms of PD patients (Temel et al., 2005). Clear delineation of the dorsoventral STN borders can help neurosurgeons to design a trajectory passing through the dorsolateral area. Furthermore, different groups have reported that the ZI is an alternative stimulation target for DBS (Ossowska, 2020). A greater therapeutic benefit for suppressing tremor in PD patients and essential tremor has been reported for the Z1 when compared to stimulation of the STN. The ZI is located cranially of the SN, dorsomedially of the internal capsule, and ventrolaterally of the RN. However, adequate visualization of the Z1 has proven to be difficult due to its small size and its location in the midbrain surrounded by different vital structures (Kerl et al., 2012). The high quality of 3-T SWI can achieve delineation of the ZI on coronal images; however, this is not possible using T2w images. We can select the electrode contact located on ZI area as the stimulation contact to improve resting tremor when we perform postoperative programming. Delineation of the ventral border of the STN on SWI is also important as this border was considered to represent the traditional depth for electrode placement. A wide variety of adverse effects, such as mania and mood disorders, are related to stimulation of the SN if the electrode is placed too deeply (Benabid et al., 2009; Raucher-Chene et al., 2008). Second, the direct targeting of the STN using SWI is a promising alternative to the use of MERs and macrostimulation. Although MER and macrostimulation can help to confirm the position of electrodes during DBS surgery, multiple microelectrode punctures may increase the risk of bleeding, infection, cerebrospinal fluid loss, and prolong the operation time. In addition, macrostimulation is usually performed by the neurosurgeon to test the therapeutic effect and side-effects, thus confirming that the electrode has been implanted in a satisfactory position. However, this technique requires the patient to cooperate in a conscious state and is not suitable for patients with surgical phobia or patients whose physical conditions cannot tolerate local anesthesia (Gross et al., 2006; Guridi et al., 2000). The direct targeting using SWI gives rise to more accurate targeting of the sensorimotor region of the STN and a larger probability of optimal electrode placement. This method can reduce the number of microelectrode punctures, reduce the occurrence of complications, and achieve asleep DBS surgery. In some patients, the bilateral STN, SN, and RN are asymmetric in their anatomical positions. The direct targeting using SWI allows neurosurgeons to asymmetrically design trajectories prior to surgery for patients with asymmetric nuclei, thus reducing the need for electrode adjustments during surgery (Schechtmann et al., 2023). In addition, the treatment of gait freezing in PD patients is a difficult problem for both neurologists and neurosurgeons. In addition to stimulating the pedunculopontine nucleus, spinal cord stimulation, low-frequency stimulation of the STN, and variable frequency stimulation of the STN, low-frequency stimulation of the SN may also improve gait freezing (Valldeoriola et al., 2019; Virmani et al., 2019). The standard electrode configuration is quadripolar, with four stimulating electrode contacts at the tip of the probe. However, it is difficult to implant electrodes simultaneously passing through the STN and SN due to length limitations. With the emergence of eight-contact electrodes, it is possible to simultaneously pass the electrodes through the STN and SN in order to improve motor symptoms and gait freezing in PD patients (Krauss et al., 2021; Thein et al., 2024). Direct targeting of the STN and SN on SWI can play an important role in preoperative planning for the application of eight-contact electrodes, visualizing contact positions after surgery, and guide postoperative programming.

Some limitations need to be noted in this study. First, the delineation of the STN borders on SWI and T2w images were compared using MER as the gold standard, but this does not mean that the results of MER-STN are the absolute true representation of STN, nor does it mean that tracks with best MER activity represent the trajectories for final electrode placement. Electrode position is also related to the results of intraoperative test stimulation. In addition, we need to consider that the fusion error of different MRI sequences will also influence the targeting accuracy. In order to reduce fusion error, high image quality is particularly important. In our center, PD patients with severe tremors were scanned while on medication or were injected with diazepam to reduce the possibility of movement artifacts during MR examinations. The fusion of all sequences was then visually inspected by two operators. The concordance of the shape and position of the grooves, anterior and posterior commissures, blood vessel courses, ventricles, midbrain outlines, and deep brain nuclei were then considered to ensure an acceptable fusion error. Through manual adjustments, the registration of SWI with T1w and T2w sequences can achieve high visual accuracy. Third, prolonged operation time, the loss of cerebrospinal fluid, pneumocephalus, and brain shift will also influence the results of MER. We opened the duramater by burning the trocar using an electrotome and injected fibrin glue to reduce the loss of cerebrospinal fluid loss and the entrance of air into the skull. Patients in this group did not experience a large amount of cerebrospinal fluid loss during surgery, and postoperative CT indicated a low incidence of the pneumocephalus. Since there are many factors that affect MER and the final position of the implantation lead, when we rely on a new MRI sequence to target the nucleus for DBS, it is necessary to use conventional targeting methods, MER, and intraoperative test stimulation to verify its accuracy to ensure the safety and effectiveness of DBS.

## 5 Conclusion

The results of our study suggested that 3-T SWI was superior in delineating the STN when compared to conventional T2w, and that the dorsoventral SWI-STN border coincided with the MER physiological border. Targeting the STN directly using SWI had a reliable accuracy (<1 mm), which was no worse than T2w. Direct targeting of the STN using SWI can help optimize preoperative target localization, trajectory planning, and postoperative programming.

## Data Availability

The raw data supporting the conclusions of this article will be made available by the authors, without undue reservation.
